# Evidence of tick-borne encephalitis virus neutralizing antibodies in Serbian individuals exposed to tick bites

**DOI:** 10.3389/fmicb.2023.1314538

**Published:** 2023-12-12

**Authors:** Pavle Banović, Dragana Mijatović, Ivana Bogdan, Verica Simin, Eleftherios Meletis, Polychronis Kostoulas, Katarina Resman Rus, Nataša Knap, Miša Korva, Tatjana Avšič-Županc, Alejandro Cabezas-Cruz

**Affiliations:** ^1^Clinic for Lyme Borreliosis and Other Tick-Borne Diseases, Pasteur Institute Novi Sad, Novi Sad, Serbia; ^2^Department of Microbiology with Parasitology and Immunology, Faculty of Medicine in Novi Sad, University of Novi Sad, Novi Sad, Serbia; ^3^Department for Research and Monitoring of Rabies and Other Zoonoses, Pasteur Institute Novi Sad, Novi Sad, Serbia; ^4^Department of Microbiology, Pasteur Institute Novi Sad, Novi Sad, Serbia; ^5^Faculty of Public and One Health, School of Health Sciences, University of Thessaly, Karditsa, Greece; ^6^Institute of Microbiology and Immunology, Faculty of Medicine, University of Ljubljana, Ljubljana, Slovenia; ^7^Anses, INRAE, Ecole Nationale Vétérinaire d’Alfort, UMR BIPAR, Laboratoire de Santé Animale, Maisons-Alfort, France

**Keywords:** ticks, TBEV, seroprevalence, TBEV-neutralizing antibodies, Serbia

## Abstract

**Introduction:**

Tick-borne encephalitis (TBE) is an emerging vector-borne and food-borne disease caused by the tick-borne encephalitis virus (TBEV; *Orthoflavivirus encephalitidis*), with a distribution spanning the Eurasian continent. Despite its significant public health impact in various European regions, TBE remains largely underdiagnosed in Serbia due to limited awareness and diagnostic challenges. In response to this, our study aimed to comprehensively assess TBEV exposure in individuals infested with ticks and to identify potential TBEV foci within Serbia.

**Materials and methods:**

From 2019 to 2021, we conducted an observational study involving 450 patients who reported tick infestations.

**Results:**

Our demographic analysis revealed a median age of 38 years, with a slight male predominance among the participants. We documented tick infestations in 38 municipalities across 14 districts of Serbia, with a notable concentration in proximity to Fruška Gora Mountain. The ticks most frequently removed were *Ixodes ricinus*, with nymphs and adult females being the predominant stages. On average, nymphs were removed after about 27.1 hours of feeding, while adult females remained attached for approximately 44.4 hours. Notably, we found age as a significant predictor of infestation time for both nymphs and adult females. Furthermore, we detected TBEV-neutralizing antibodies in 0.66% of the serum samples, shedding light on potential TBEV foci, particularly in Fruška Gora Mountain and other regions of Serbia.

**Conclusion:**

Our study emphasizes the urgent need for active TBE surveillance programs, especially in areas suspected of hosting TBEV foci, in order to assess the true TBE burden, identify at-risk populations, and implement effective preventive measures.

## 1 Introduction

Tick-borne encephalitis (TBE) is an emerging vector-borne and food-borne disease caused by the TBE virus, a member of the *Orthoflavivirus* genus, Flaviviridae family (Elbaz et al., 2022; Kunze et al., 2022; Postler et al., 2023). This virus is distributed across the Euroasian continent, forming a “TBE belt” stretching from Western Europe to the easternmost regions of Asia (Kunze et al., 2022). TBEV is classified into five subtypes based on antigenic characteristics, disease severity, and geographic distribution: European (TBEV-Eu), Siberian (TBEV-Sib), Far Eastern (TBEV-Fe), Baikalian (TBEV-Bkl), and Himalayan (TBEV-Him; Dai et al., 2018).

Tick-borne encephalitis-European primarily infects humans through the bites of infected *Ixodes ricinus* tick and occasionally via the consumption of unpasteurized milk products contaminated with TBEV. This European subtype is prevalent in Western, Central, and Southeastern Europe and is generally less virulent compared to TBEV-Sib and TBEV-Fe. Consequently, it is associated with milder forms of central nervous system (CNS) infections, more favorable outcome, and fewer long-term complications in survivors. Nonetheless, TBE remains a significant public health concern, particularly in Central and North-eastern Europe, with the highest number of cases reported in the Baltic countries, Czechia, Switzerland, Slovakia, Slovenia, Austria, and Germany (Steffen, 2016; Kunze et al., 2022).

Following exposure to TBEV, individuals can develop subclinical, abortive, or clinical infections. It is estimated that the majority of TBEV-Eu infections are subclinical, while approximately 30% progress to the febrile stage (Stage I) with concurrent viremia. Among these, 70% will clear the virus and have an abortive infection, while the remaining 30% will experience CNS involvement and develop manifest TBE (Barp et al., 2020; Bogovič et al., 2022a,b).

Due to the short viremia period and non-specific disease signs during Stage I (i.e., most patients seek medical attention when neurological symptoms appear), the definitive diagnosis of TBE relies on indirect methods, and most commonly enzyme-linked immunosorbent assays (ELISAs; Kunze et al., 2022). In regions where both TBEV and West Nile virus (*Orthoflavivirus nilense*; WNV) infections occur, such as Serbia, Hungary, Croatia, and Romania (Popescu et al., 2018; Zana et al., 2020; Petrović et al., 2021), ELISA results may lead to misdiagnosis of TBE as West Nile encephalitis and vice versa due to antibody cross-reactivity. Therefore, neutralization assays are often necessary for accurate differentiation (Potkonjak et al., 2017; Kunze et al., 2022).

Despite TBE being a notifiable disease in Serbia since 2004, there is limited awareness of the disease among medical practitioners (Vasić et al., 2022). The most recent detection of TBEV in ticks in Serbia dates back to 2017 (Potkonjak et al., 2017). Additionally, TBEV-reactive antibodies have been recently detected in individuals recovering from viral meningitis/encephalitis of unknown origin and in tick-infested patients (Banović et al., 2021, 2022a). Until recently, there was no active TBE surveillance program focused on serological or molecular screening of patients with CNS infection symptoms, and the assay for detecting TBEV-neutralizing antibodies (TBEV-NAb) was not available (Popović Dragonjić et al., 2022).

To assess the extent of TBEV exposure in the Serbian population and identify risk groups and high-incidence areas, we conducted a prospective observational study between 2019 and 2021. This study aimed to determine the prevalence of TBEV-NAbs in individuals who had been infested with ticks.

## 2 Materials and methods

### 2.1 Study design and participant enrollment

This observational study leveraged medical records obtained during routine operations at the Clinic for Lyme Borreliosis and Other Tick-Borne Diseases, Pasteur Institute Novi Sad (PI Novi Sad) between January 1, 2019 and December 31, 2021. Serum samples were collected from patients with tick infestations. The medical records included basic demographic information (age, gender), tick-borne disease development, and data related to the ticks removed from patients (e.g., species, life stage, and feeding duration). Serum samples were taken at least 4 weeks after tick removal from patients that met the inclusion criteria and provided informed consent, for the detection and quantification of TBEV-neutralizing antibodies. Additionally, samples showing neutralizing effect to TBEV were examined for WNV-neutralizing antibodies to rule out cross-reactivity.

To be eligible for enrollment and serum sampling, patients needed to meet the following criteria: (i) patients who reported to PI Novi Sad with ticks still attached or recently removed and submitted them for entomologic examination; (ii) patients who underwent a total of three medical examinations during a 2-month follow-up period, including a visit for blood sampling at least 4 weeks after tick removal; (iii) patients or their caregivers provided informed consent for study inclusion, medical record analysis, and blood sampling; and (iv) patients had not been immunized against TBE or yellow fever.

### 2.2 Tick collection and classification

All ticks collected from patients were identified by species, sex, and life stage based on morphological features and standard taxonomic keys as described by Estrada-Peña (2004). Patients also provided information about where they suspected they acquired the ticks. When patients were uncertain about the time between tick bite and detection, attachment time was estimated from approximate feeding time, assessed using the coxal index (for tick attachments lasting <24 h) and the scutal index (for tick attachments lasting 24 h or more; Gray et al., 2005). Since feeding time assessed by measurement of scutal or coxal index is expressed in hours, we divided final values by 24 and expressed infestation time as number of days.

### 2.3 Blood sample collection and sera extraction

At least 4 weeks after tick removal and with informed consent obtained from each patient or their caregivers (for underage individuals), a 3 mL blood sample was collected using BD Vacutainer® SST™ Tubes (BD, Franklin Lakes, NJ, United States). The blood samples were allowed to clot at room temperature, followed by centrifugation at 2,000 × g for 10 min to extract and store the serum at −80°C until further analysis.

### 2.4 Detection of TBEV-Nabs

The TBEV strain Neudörfl (National Collection of Pathogenic Viruses, United Kingdom; Cat. No 0201139v) was cultured in a biosafety level 2+ Laboratory for Vector Borne Pathogens at Pasteur Institute Novi Sad using a monolayer of BHK-21/C13 cells (BS CL 8, Istituto Zooprofilattico Sperimentale Brescia, Italy). Virus stocks were prepared at a concentration of 100 Tissue Culture Infectious Dose (TCID)/100 μL and stored at −80°C. The micro-neutralization test (micro-NT) was conducted using a 96-well cell culture plate (Thermo Scientific™, MA, United States, Cat. No 130338). Serum samples were first inactivated at 56°C for 30 min and tested in duplicate with serial dilutions ranging from 1:5 to 1:40 in Glasgow Minimal Essential Medium (Biowest, France; Cat. No P0120). Each test run included positive and negative controls, a cell control, and virus back-titration. A total of 100 TCID of virus stock was added to the respective serum dilutions and incubated for 1 h at 37°C. After incubation, the serum-virus mixture was transferred to wells containing BHK21/C13 cells seeded at a concentration of 2 × 10^5^ cells and incubated for 5 days at 37°C with 5% CO2. The cytopathic effect (CPE) was observed in both wells for each sample. The dilution of the sample resulting in virus neutralization in 50% of the replicates (NT_50_) was calculated using the Spearman and Karber method (Ramakrishnan, 2016) and represented as the reciprocal value of the same dilution.

### 2.5 External validation of TBEV neutralization assay

The TBEV neutralization assay was performed in a biosafety level 3 facility. First, Vero E6 cells (ATTC CRL-1586) were seeded at 10^5^ cells/well in 96-well plates (TPP, 92196) supplemented with growth medium consisting of DMEM with GlutaMAX supplement (Thermo Fisher Scientific, 61965026) and 10% FBS (Euroclone, ECS0180L) and incubated for 24 h at 37°C in 5% CO2. Two-fold serial dilutions (initial dilution was 1:10) of heat-inactivated sera samples (56°C, 30 min) were incubated with 100 TCID_50_ of TBEV (TBEV strain Ljubljana 1; deposited in the EVA-GLOBAL Virus Archive under reference number Ref-SKU: 007 V-EVA71) for 1 h at 37°C. Then, 50 μL of the serum dilution-virus mixture was inoculated in triplicate into a 96-well plate containing an 80% confluent Vero E6 monolayer. The plates were incubated for 5 days at 37°C before being fixed with 50 μL of 4% formaldehyde. Plates were then examined microscopically for cytopathic effect (CPE) and the cell monolayer was additionally visualized by crystal violet staining. The neutralization endpoint titer was defined as the endpoint plasma dilution that inhibited TBEV-induced cytopathic effect in at least two out of three parallels. Positive and negative control sera were included in each plate.

### 2.6 Detection of cross-reactivity via West Nile virus neutralization assay

To exclude the possibility of antibody cross-reactivity, we also performed a West Nile virus neutralization assay using 100 TCID_50_ of WNV (strain: Eg101; deposited in the EVA-GLOBAL Virus Archive under reference number Ref-SKU: 007 V-03213) according to the protocol described in Knap et al. (2020).

### 2.7 Bias identification and management

The most significant bias identified in this clinical observational study is the proximity of our center to patients’ homes. Patients residing in urban and suburban areas of Novi Sad are more likely to seek medical attention at PI Novi Sad compared to those who need to travel longer distances for follow-up examinations. To address this bias, general practitioners across the Vojvodina autonomous province (North Serbia) were instructed to encourage patients to visit PI Novi Sad regardless of whether they acquired ticks within or outside their home city. This approach allowed us to analyze ticks from various locations across Serbia, not limited to patients’ municipalities of residence.

### 2.8 Statistical analysis

In this study, we employed the Cox proportional hazards mixed-effects model, referred to as the Cox mixed-effects model, to analyze the time taken until tick removal, which was the event of interest (dependent variable). For all patients, a tick was removed, and thus, the event was coded as 1 for each individual. The primary objective of the analysis was to identify the most influential predictors for the time until tick removal. The independent variables examined were demographic information, including age, gender, and place of residence. We conducted separate analyses for nymphs and adult females. Additionally, to visually depict the time until removal for specific clusters, such as age groups divided into 10-year intervals and gender, Kaplan–Meier survival curves were constructed. The analysis and visualization were performed using the R programming language (R-Project, 2023).

Regarding the number of seroreactive samples, the data analysis was limited to descriptive statistics of laboratory findings. Fisher’s exact test was used to assess differences in demographic characteristics within the examined cohort, with statistical significance considered for *p* < 0.05. Statistical analysis and visualization were conducted using GraphPad software v.9 (GraphPad Software Inc., La Jolla, CA, United States).

## 3 Results

### 3.1 Ethics statement

This study was approved by the ethical committee of Medical faculty Novi Sad, University of Novi Sad (Ethical approval No. 01-39/261/1 from 17.09.2020) and conducted according to the Declaration of Helsinki and The Patient Rights Law of the Republic of Serbia.

### 3.2 Participants enrollment

**Figure 1:**
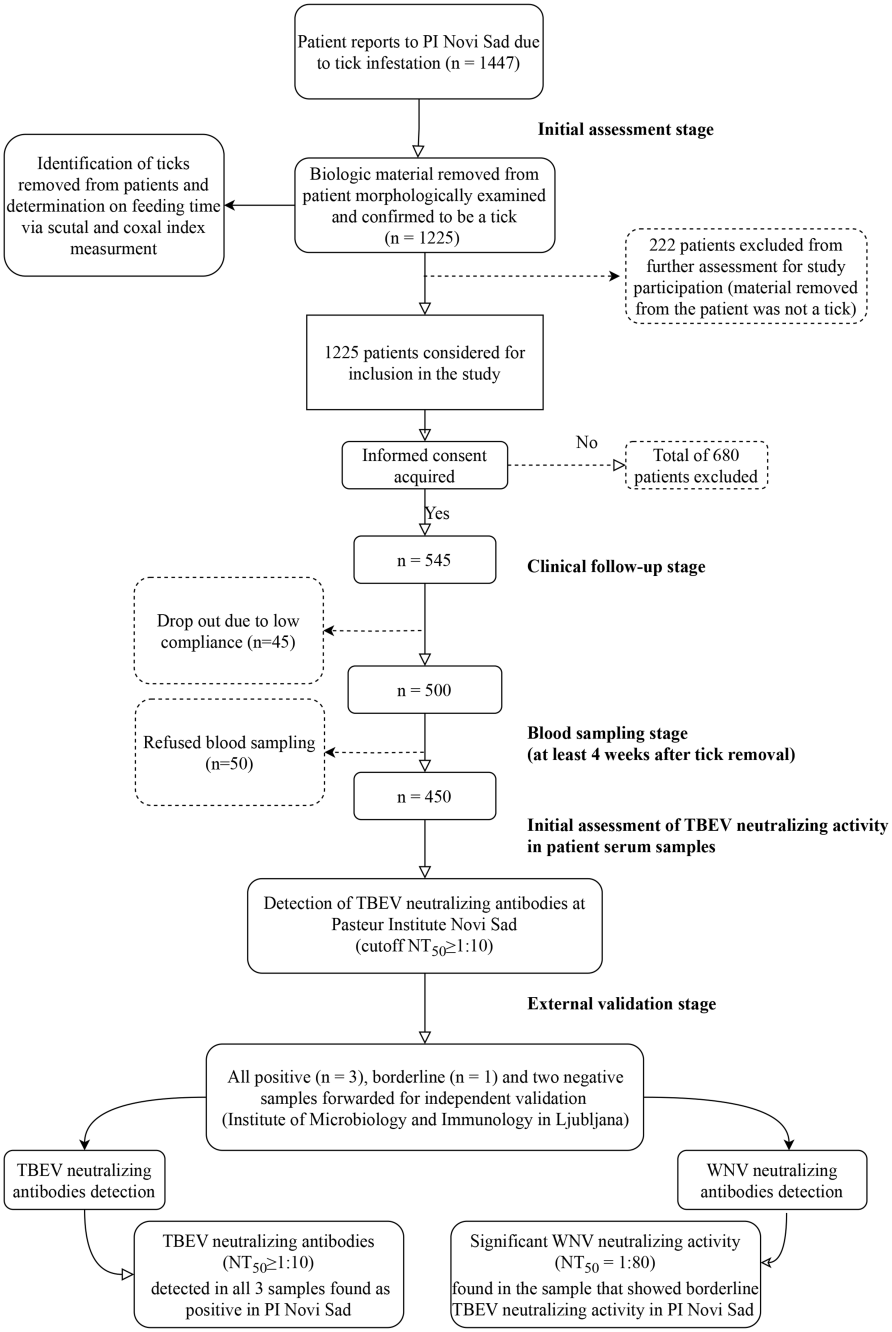
Study flowchart. PI Novi Sad, Clinic for Lyme borreliosis and other Tick-Borne Diseases of the Pasteur Institute Novi Sad; TBEV, Tick-borne encephalitis virus; WNV, West Nile virus; NT_50_, titer that neutralizes 50% of challenge virus. Flowchart generated using open-source software draw.io (https://app.diagrams.net/).

Between January 1, 2019 and December 31, 2021, a total of 1,447 patients sought medical consultation at PI Novi Sad, reporting tick bites and tick-associated infections. To be eligible for study inclusion, patients needed to either bring the tick removed from their skin or present with an attached tick. Out of these patients, 1,225 were confirmed to have been exposed to tick bites, while 222 individuals reported with various biologic materials of different origins, such as fabric fragments, crusts, small warts, spiders, or coagulated blood. Informed consent for study enrollment was obtained from 545 individuals (44.48%) among those confirmed to be exposed to tick bites. However, during the 4-week follow-up period, 45 participants dropped out due to low compliance, and an additional 50 patients refused to have their blood sampled after the follow-up period. Ultimately, the cohort of tick-exposed patients comprised 450 individuals who completed the observation period and underwent blood sampling (Figure 1).

### 3.3 Characteristics of enrolled patients and tick exposure locations

Demographic analysis revealed that the median age of tick-infested individuals included in the study was 38 years (interquartile range: 10–58 years). In terms of gender, males constituted a non-significant majority (237/450; 52.66%, Fisher exact test value 0.4632; *p* > 0.05).

**Figure 2:**
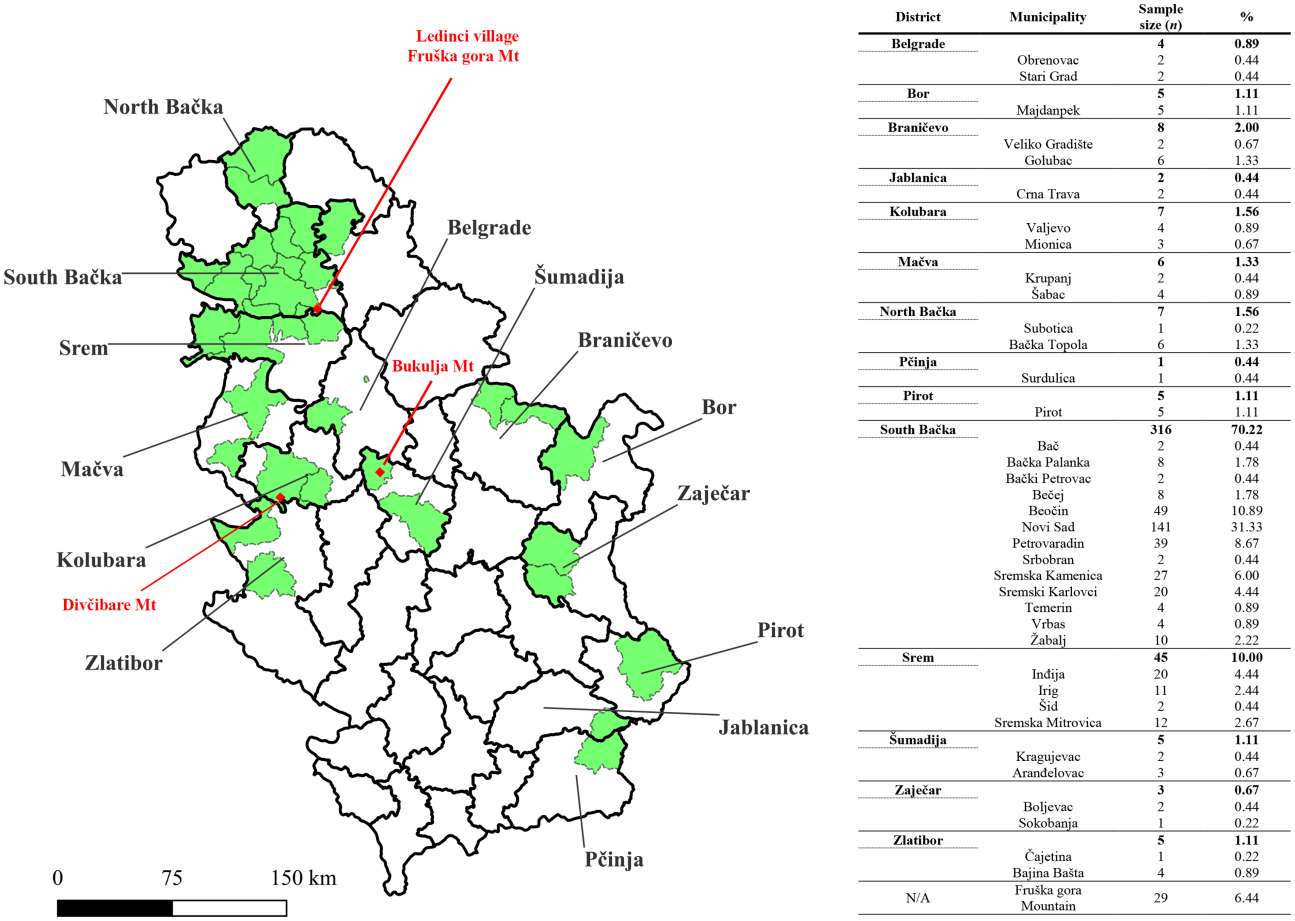
Map of Serbia showing Geographical locations where tick infestations occurred in patients reported to Pasteur Institute Novi Sad. The sample distribution is presented by municipalities (green color), across 14 districts of Serbia. Red dots are used to mark locations where tick infestation occurred for three patients whose serum had TBEV-neutralizing capability. Mt., Mountain. The shape file for mapping at district and municipality levels is available at the GADM database of Global Administrative Areas (v4.1, July 2022; https://gadm.org/). The map was generated by using QGIS v3.12 (QGIS Development Team 2020).

An epidemiological survey, supplemented by determination of tick feeding time, indicated that tick infestations occurred in 38 municipalities spanning 14 districts across Serbia (Figure 2). Tick infestations were most frequent in municipalities proximate to Fruška Gora Mountain, particularly within the Srem and South Bačka districts.

### 3.4 Characteristics of ticks removed from patients, assessment of tick attachment time

*Ixodes ricinus* accounted for the overwhelming majority of ticks removed from patients (432/450; 96%). Other tick species, such as *Dermacentor reticulatus* (4/450; 0.88%, all adult females), *Dermacentor marginatus* (4/450; 0.88%, all adult females), *Rhipicephalus sanguineus* (6/450; 1.33%, two adult males and four adult females), and *Haemaphysalis punctata* (4/450; 0.88%, all adult females), were observed less frequently.

Among *I. ricinus* ticks, nymphs (235/432; 54.39%) and adult females (184/432; 42.59%) were the most prevalent. Infestations caused by larvae and adult males were rare (11/432; 2.54% and 2/432; 0.46%, respectively). On average, nymphs were removed after approximately 27.1 h of feeding (95% CI 38–49.2), while adult females achieved infestations lasting approximately 44.4 h (95% CI 33.84–54.96).

**Figure 3:**
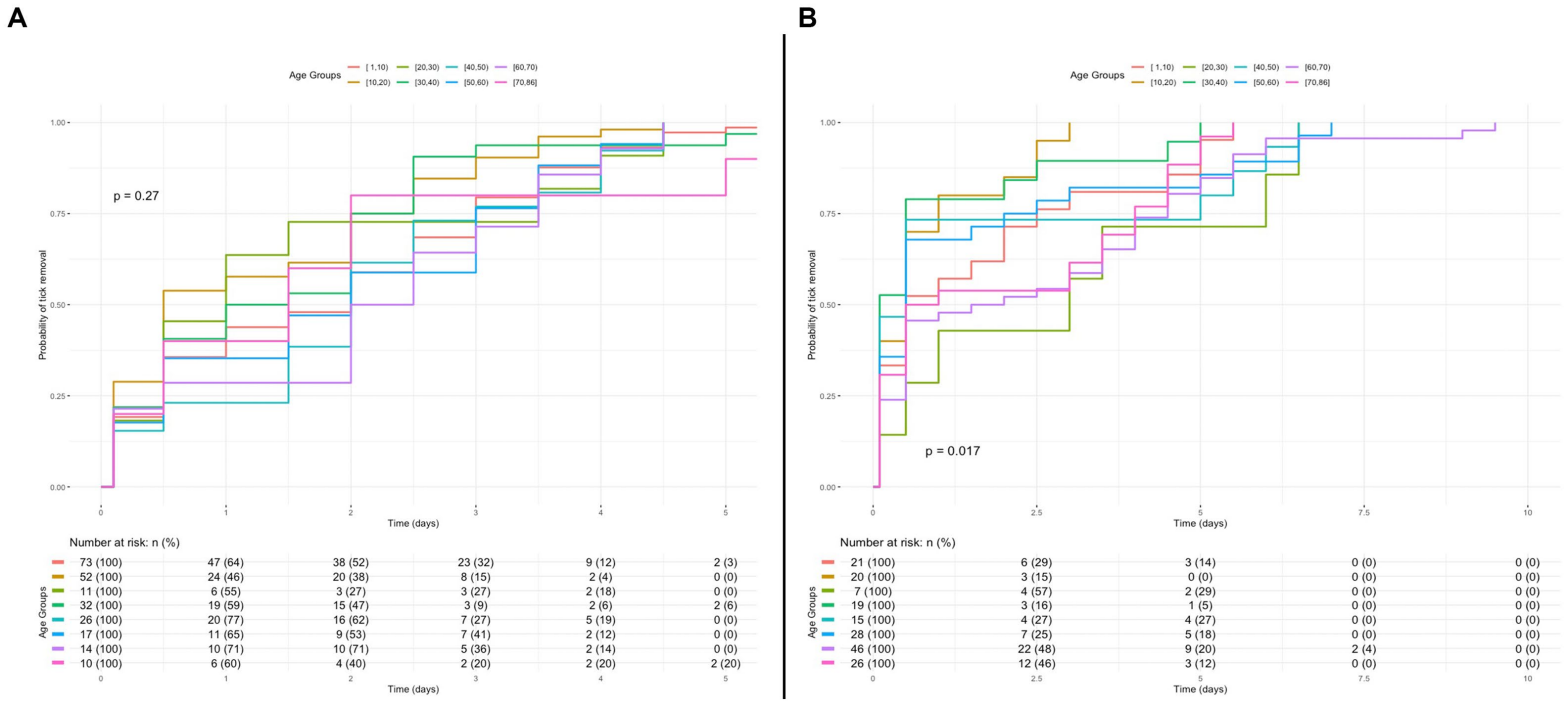
Kaplan–Meier curve of probability for removal of *Ixodes ricinus* removal in the course of time for different age groups. **(A)** Kaplan–Meier curve for probability of *I. ricinus* nymph removal in the course of time for the different patient age groups of the study. A *p* value higher than 5% indicates no statistically significant difference between groups. **(B)** Kaplan–Meier curve for probability of *I. ricinus* adult female removal in the course of time for the patient different age groups of the study.

The Cox proportional hazards mixed model identified age as a parameter associated with the duration of *I. ricinus* nymph feeding. Specifically, the age group of 10–20 years exhibited a statistically significant relationship with the outcome (i.e., infestation period assessed by scutal/coxal indices). The reported odds ratio was 1.54 (95% CI 1.06–2.22), with a value of *p* of 0.022, suggesting that patients aged 10–20 years may have a distinct relationship with the shorter time required to remove infesting ticks when compared to the reference level or other age groups (Figure 3A).

The Cox proportional hazards mixed model for the infestation time of adult females showed a *p* value of <0.05, indicating the possibility of one or more age groups differing from the others (Figure 3B). Further investigation through pairwise comparisons of Kaplan-Mayer curves revealed a statistically significant difference between the age group of 10–20 years and the age group of 60–70 years (*p* = 0.014), where the age group of 10–20 years was associated shorter infestation period, despite this not being captured or reported in the model.

### 3.5 Prevalence of TBEV-neutralizing antibodies

Out of the 450 serum samples examined at PI Novi Sad, TBEV NT_50_ ≥ 10 was observed in three samples, with one sample recording NT_50_ = 5, categorized as borderline. Subsequent analysis conducted at the Institute of Microbiology and Immunology, Faculty of Medicine, in Ljubljana (University of Ljubljana, Slovenia) confirmed the presence of TBEV-neutralizing antibodies in all three samples initially identified as positive in PI Novi Sad, with no neutralization effect against WNV. Additionally, the sample initially categorized as borderline (TBEV NT_50_ = 5) exhibited a significant neutralization effect against WNV (WNV NT_50_ = 80), with no neutralization effect against TBEV, ultimately being classified as negative for TBEV-neutralizing antibodies. Three samples initially identified as negative in PI Novi Sad were confirmed to be absent of TBEV-neutralizing antibodies as well as WNV-neutralizing antibodies. In summary, the presence of TBEV-neutralizing antibodies was confirmed in three serum samples (3/450; 0.66%), with NT_50_ values of 10, 160, and > 1,280. None of the patients with TBEV-neutralizing antibodies exhibited signs of stage I or II TBEV infection, and denied previous immunization against TBE and yellow fever, as well as medical history of aseptic meningitis and/or encephalitis. All three individuals were infested with *I. ricinus* ticks in 2021 (one larva, one nymph, and one adult female, respectively). The suspected locations of tick infestation for the three seropositive patients were Ledinci village (Fruška Gora Mountain, Municipality of Novi Sad), Divčibare Mountain (Municipality of Valjevo), and Bukulja Mountain (Municipality of Aranđelovac; Figure 2).

## 4 Discussion

Tick-borne encephalitis virus foci are intricate microecosystems heavily influenced by a multitude of biotic and abiotic factors that can either facilitate or hinder virus transmission and the amplification chain (Cabezas-Cruz and Banović, 2023). The European continent is currently witnessing the expansion of TBEV endemic regions, primarily driven by climate changes, various anthropogenic factors, and the resulting migration of animals that serve as tick hosts (Kunze et al., 2022). Variation in these factors can result in disappearance of existing TBEV foci when the maintenance and transmission of the virus between vectors (i.e., *Ixodidae*) and reservoirs (e.g., small rodents of the genera *Apodemus* and *Myodes*) are disturbed (Knap and Avšič-Županc, 2015; Bournez et al., 2020). As TBEV foci expand, the incidence of TBE is on the rise across Europe, even in higher-altitude regions of Central Europe and previously considered TBEV-free areas such as Scandinavian countries (Kunze et al., 2022).

Despite TBE being a notifiable disease in Serbia, reported cases are infrequent due to low awareness among healthcare workers (Vasić et al., 2022). Another challenge in TBE diagnostics has been the absence of a gold standard diagnostic assay, as the detection of TBEV-neutralizing antibodies in Serbia only became possible in 2022 (Popović Dragonjić et al., 2022). Unlike many European countries where concerns over TBEV are growing, Serbia lacks an active surveillance program to assess the prevalence of TBEV infection in ticks and/or rodents. The last detection of TBEV in Serbia occurred in 2017 when infected *I. ricinus* ticks were collected in various locations within the suburbs of Belgrade and Fruška Gora Mountain (Potkonjak et al., 2017).

The existence of TBEV foci in different regions of Serbia was evident when tick-infested patients exhibited a higher likelihood of seroreactivity to TBEV antigens compared to blood donors from the same region (Banović et al., 2021). Further awareness arose when TBEV-reactive IgG were discovered in 20% of individuals recovering from viral encephalitis of unknown origin (Banović et al., 2022a; Cabezas-Cruz and Banović, 2023). However, a significant limitation of these studies is that TBEV exposure was inferred through assays not designed to differentiate anti-TBEV antibodies from the humoral response generated against other orthoflaviviruses circulating in Serbia (e.g., *Orthoflavivirus usutuense* and WNV). Indeed, when a neutralization assay was applied in a two-center seroprevalence study conducted in Novi Sad (Serbia) and Skopje (North Macedonia), no TBEV-neutralizing antibodies were found in the Serbian cohort exposed to tick bites (Jakimovski et al., 2023). This result may be influenced by the small number of participants included in the Serbian cohort (*n* = 51), compared with the cohort examined in this study (*n* = 450).

Among the 450 individuals exposed to tick bites, TBEV-neutralizing antibodies were found in three patients, representing a seroprevalence of 0.66%. This seroprevalence is considerably lower than the prevalence of IgG reactivity to TBEV in tick-infested individuals from Serbia (13.27%; Banović et al., 2021). This discrepancy is expected, as several members of genus *Orthoflavivirus* are circulating within Serbian population and causing development of specific immunoglobulins (Petrović et al., 2019, 2021) that can cross-react with other members of the *Orthoflavivirus* genus. In addition, TBEV-Nabs are only a subset of total anti-TBEV antibodies generated after exposure to TBEV. Although, TBEV-Nab seroprevalence in our cohort is higher compared to Norwegian, Romanian and Serbian blood donors (0.66 vs. 0.4% and 0.08 and 0%, respectively; Marvik et al., 2021; Coroian et al., 2022; Jakimovski et al., 2023) and much lower compared to residents of Czechia (0.66% vs. 26.3%; Kriz et al., 2015).

To identify potential TBEV foci in Serbia, we integrated anamnestic data and an epidemiological survey with scutal/coxal index values of ticks removed from patients with TBEV-neutralizing antibodies. We identified potential locations within three mountains: Fruška Gora Mountain, Divčibare Mountain, and Bukulja Mountain. Among these, Fruška Gora Mountain is the only locality previously suspected in seroprevalence and field studies to have an environment conducive to the maintenance of TBEV foci (Potkonjak et al., 2017; Banović et al., 2021). Regarding the tick species responsible for human infestations, our finding that *I. ricinus* was the most frequently removed tick aligns with previous studies conducted in Serbia (Banović et al., 2022b, 2023). Although *I. ricinus* ticks were removed from all three patients in whom TBEV-neutralizing antibodies were detected, we are not able to claim that TBEV exposure occurred during most recent infestation. Concerning the infestation time, the significance of age as a predictor level and the output of the Cox model used here necessitate further investigation. When a level of a predictor variable (age group) in a Cox proportional hazards model has a *p* value greater than 5%, it suggests that overall, that specific age group might not have a statistically significant difference in infestation time compared to other age groups. However, when conducting pairwise comparisons among levels of the predictor (i.e., age groups), it might be found that one particular age group significantly differs from another, even if its overall relationship in the main model was not statistically significant (Hosmer et al., 2011; Agresti, 2012).

A major limitation of this study is the absence of attempts to isolate and/or detect TBEV in ticks removed from humans, which could confirm recent exposure to the virus in patients with TBEV-neutralizing antibodies. Additionally, a 4-week period between tick infestation and serum sampling may not be long enough for all TBEV-exposed persons to develop detectable levels of TBEV-neutralizing antibodies. Uneven distribution of samples across the country limits the representativeness of the results for all districts of Serbia, necessitating further studies where several centers will be engaged in serum collection, where period between tick infestation and serum sampling should be expanded to at least 5 weeks. Nevertheless, this study is the first to provide unequivocal evidence of TBEV exposure in tick-infested individuals from Serbia. Since anti-TBEV IgM reactivity and anti-TBEV IgG avidity were not determined, we are not able to claim that most recent (i.e., observed) tick infestation is linked with virus exposure in patients with TBEV-neutralizing antibodies. Therefore, infestation locations should be considered as potential natural foci, warranting future surveillance campaigns. Healthcare institutions should initiate surveillance programs and test all cases of viral meningitis/encephalitis of unknown origin for the presence of TBEV-neutralizing antibodies, especially those responsible for the residents of Fruška Gora, Divčibare, and Bukulja mountains. This approach will enable the assessment of the TBE burden in the Serbian population, identification of risk groups, field research, and the implementation of prevention measures, including immunization and educational campaigns.

## 5 Conclusion

In this study, we have unveiled critical insights into the seroprevalence of TBEV-neutralizing antibodies and epidemiological significance of TBEV in Serbia. Although we were not able to provide the evidence of recent infection in tested patients, our findings suggest the existence of potential TBEV foci within Serbia, notably in regions proximal to Fruška Gora Mountain, Divčibare Mountain, and Bukulja Mountain. The detection of TBEV-neutralizing antibodies in tick-infested individuals, albeit at a relatively low prevalence of 0.66%, underscores the need for heightened surveillance efforts. The identification of TBEV exposure in these regions highlights the urgency of implementing active TBE surveillance programs. Furthermore, our study has shed light on the demographics of tick-infested individuals and the characteristics of tick infestations, including attachment times of different tick stages. Age was identified as a significant predictor of infestation time, emphasizing the importance of considering age-related factors in future TBE research and surveillance. Research efforts as presented in this paper are crucial for assessing the true burden of TBE, identifying high-risk groups, conducting further field research, and ultimately implementing effective prevention measures to mitigate the spread of this emerging disease.

## Data availability statement

The original contributions presented in the study are included in the article/supplementary material, further inquiries can be directed to the corresponding author.

## Ethics statement

This study was approved by the ethical committee of Medical faculty Novi Sad, University of Novi Sad (Ethical approval No. 01-39/261/1 from 17.09.2020.) and conducted according to the Declaration of Helsinki and The Patient Rights Law of the Republic of Serbia. The studies involving humans were approved by Komisija za etičnost kliničkih ispitivanja Medicinskog fakulteta u Novom Sadu. The studies were conducted in accordance with the local legislation and institutional requirements. Written informed consent for participation in this study was provided by the participants’ legal guardians/next of kin. The manuscript presents research on animals that do not require ethical approval for their study.

## Author contributions

PB: Conceptualization, Formal analysis, Funding acquisition, Investigation, Methodology, Project administration, Validation, Visualization, Writing – original draft, Writing – review & editing. DM: Formal analysis, Investigation, Methodology, Writing – original draft. IB: Conceptualization, Investigation, Methodology, Writing – original draft. VS: Conceptualization, Data curation, Investigation, Methodology, Writing – review & editing. EM: Formal analysis, Investigation, Methodology, Software, Visualization, Writing – review & editing. PK: Investigation, Methodology, Software, Supervision, Visualization, Writing – review & editing. KR: Formal analysis, Investigation, Methodology, Validation, Writing – review & editing. NK: Investigation, Methodology, Validation, Writing – review & editing. MK: Data curation, Investigation, Resources, Validation, Writing – review & editing. TA-Ž: Conceptualization, Methodology, Resources, Supervision, Validation, Writing – review & editing. AC-C: Data curation, Formal analysis, Methodology, Supervision, Writing – review & editing.
